# Identification of immunopeptides (pHLA) as candidate therapeutic targets in chondrosarcoma

**DOI:** 10.1016/j.jbo.2026.100780

**Published:** 2026-07-02

**Authors:** Léa Rogue, Jean-Marc Monneuse, Céline Béchon, Lola Cepero, Caroline Peyrode, Sandrine Viala, Maud Privat, Yannick Bidet, Adrien Saliou, Paul-Olivier Rouzaire, Elisabeth Miot-Noirault, Florent Cachin, Aurélien Pommier

**Affiliations:** a UMR1240 Imagerie Moléculaire et Stratégies Théranostiques INSERM, Université Clermont Auvergne, BP 184, F-63005 Clermont-Ferrand, France; b BIOASTER, Microbiology Technology Institute, F-69007 Lyon, France; c Centre Jean Perrin, F-63000 Clermont-Ferrand, France; d CHELTER, CHU Clermont Ferrand, F-63000 Clermont-Ferrand, France

**Keywords:** Chondrosarcoma, Cancer testis antigens (CTAs), Immunotherapy, Immunopeptidome, T cell therapies

## Abstract

**Background:**

Chondrosarcoma (CHS) is the second most common primary malignant bone tumor and remains resistant to conventional therapies, underscoring the need to discover novel therapeutic targets. Cancer/testis antigens (CTAs), a class of tumor-associated proteins, represent attractive antigens for cancer immunotherapies such as adoptive T cell therapy. However, the expression profile of CTAs and their associated targetable immunopeptides presented in the Human Leukocyte Antigen-I context (pHLA) remain unknown in CHS. This study aims to characterize the CTA expression profile according to the tumor immune phenotype and clinical outcomes, and to identify the most relevant pHLA to target in CHS.

**Method:**

We analyzed the CTA expression profile in tumors from 63 conventional CHS patients and in healthy tissues using GTEx and HPA databases to identify CHS-associated CTAs. Cox proportional hazards models combined with hierarchical clustering were used to correlate CTA expression with the overall survival of patients. The tumor immune phenotype was estimated based on immune gene expression signatures using a deconvolution method and a Pearson correlation coefficient matrix. The CTA-derived pHLA were characterized using immunopeptidomic profiling based on HLA-I immunoprecipitation and mass spectrometry in grade 2 and 3 CHS models. NetMHC was used to predict the binding affinity of identified pHLA to the HLA-A*02:01 and HLA-A*01:01 alleles.

**Results:**

We identified a poor prognosis CTA signature predominantly associated with a non-inflamed tumor immunophenotype. Immunopeptidomic profiling revealed broad pHLA repertoires, including previously well-characterized CTAs from PRAME, CTAG2 and the MAGE-A family, as well as newly identified candidates with strong predicted HLA-binding affinity from CTAs such as HHIPL2, DBF4, BRIP1, CBX2 and DIAPH3.

**Conclusion:**

This study provides the first atlas of pHLA in CHS and suggests specific antigenic targets for TCR-T cell therapies and targeted therapies such as antibody-drug conjugates.

## Highlights

• >7000 novel pHLA were identified including 175 original peptides derived from CTAs.
• Well-characterized pHLA from PRAME, CTAG2 or MAGE-A family may be exploitable in CHS.
• Novel pHLA were identified from clinically relevant CTAs including DBF4, CBX2, DIAPH3.
• Poor prognosis CTAs are associated with non-inflamed tumor immunophenotype in CHS.

## 1 Introduction

Chondrosarcoma (CHS) is the second most prevalent primary malignant bone tumor after osteosarcoma, with an estimated global incidence of approximately 1 per 200,000 individuals annually. [1] Conventional CHS represents approximately 90% of all CHS cases and is histologically classified into grades 1–3, which closely correlate with metastatic risk and clinical outcomes. [1], [2] Owing to their abundant extracellular matrix, low proliferative activity, and limited vascular supply, these tumors demonstrate marked resistance to both chemotherapy and radiotherapy [3], [4] Surgical intervention including tumor excision or, in some cases, amputation, remains the only potentially curative treatment. However, CHS remains highly invasive and is associated with substantial morbidity, with a 5-year survival rate below 50% for the most aggressive forms. [1], [5] The 10-year survival rate varies widely, ranging from 29% to 83%, depending on tumor subtype and histologic grade. [1], [6], [7] Therefore, the development of alternative non-surgical therapeutic options is urgently needed, particularly for individuals with unresectable, metastatic, or recurrent tumors.

Immunotherapy has demonstrated significant efficacy across multiple malignancies, including melanoma and lung cancer. [8], [9], [10] However, in the context of musculoskeletal sarcomas such as CHS or osteosarcoma, its application remains at an early stage of development. The approval of the immune-stimulating agent mifamurtide by the European Medicines Agency for the treatment of osteosarcoma, along with the implementation of adoptive T cell therapy in synovial sarcoma [11], represent two promising initial steps toward integrating immunotherapy into sarcoma management. Despite these advances, the most relevant tumor-associated antigens, which may represent therapeutic targets for adoptive T cell therapy, have not been widely explored in CHS. Due to their tumor-associated expression, cancer/testis antigens (CTAs) represent promising targets for cancer-specific immunotherapies.

CTAs are a group of developmentally regulated proteins mainly restricted to male germ cells but aberrantly re-expressed in various cancers, where they can elicit immune responses and are therefore classified as tumor-associated antigens. Their expression is heterogeneous and often correlates with tumor progression. [12] CTAs have been identified in multiple malignancies, including melanoma, liver, lung, and bladder cancers, as well as pediatric tumors such as neuroblastoma. [13], [14], [15] During gametogenesis, they contribute to germ cell differentiation and proliferation. [16]

This study aims to explore which CTAs are the most relevant to target in conventional CHS and to identify the CTA-derived Human Leukocyte Antigen peptides (pHLA) by combining transcriptomic and immunopeptidomic analyses from CHS patients and human CHS cell lines.

## 2 Materials and methods

### 2.1 Public datasets

The list of CTAs was defined based on the CTDatabase (http://www.cta.lncc.br/) which contains confirmed CTA genes and additional putative CTAs proposed by Wang et al. [17] Transcriptomic microarray data from CHS were retrieved from Nicolle et al. (E-MTAB-7264), which include 63 conventional CHS samples with clinical and pathological information (overall survival and tumor stage). [18] Open-access RNA-seq data from the Genotype-Tissue Expression (GTEx) project and the Human Protein Atlas (HPA) were used to assess CTA expression across healthy tissues. For each gene, transcripts per million (TPM) values were averaged per tissue to generate a reference atlas of CTA expression in normal tissues.

Immune infiltration was estimated using the gene signature defined by Bindea et al., which includes marker sets for major immune cell types. [19] For each signature, the mean expression of its constituent genes was computed to infer the relative infiltration level of immune cell populations.

Known human pHLA were retrieved from the Immune Epitope Database (IEDB, downloaded 23/09/2025). These data were used to annotate peptides from the immunopeptidomic dataset, identify novel peptides derived from CTAs, and support peptide immunogenicity predictions.

### 2.2 Cell line cultures

The human CHS cell line (JJ012) was provided by Joel A. Block, M.D., Rush Medical College, Rush University Medical Center, Chicago, USA. The Human CHS CH2879 cell line was provided by Yannick Saintigny, LARIA, CEA Caen. JJ012 (grade 2) and CH2879 (grade 3) are both primary central CHS cell lines isolated from a 39-year-old male patient and a 35-year-old female patient, respectively. Both cell lines were cultivated in RPMI 1640 (Gibco) supplemented with 10% fetal bovine serum (Dutscher), 1% penicillin-streptomycin (Gibco) and maintained at 37 °C in a humidified atmosphere with 5% CO_2_. IFNγ treatment (Biolegend) was performed 24 h post-seeding for 3 days. Cells were harvested at 80% confluence for transcriptomic and immunopeptidomic analyses.

### 2.3 Transcriptomic analyses

Total RNA was extracted from fresh cell cultures using the RNeasy kit (Qiagen). RNA quality was verified on a TapeStation 4150 system using High Sensitivity RNA ScreenTapes (Agilent®), ensuring an RNA Integrity Number (RIN) greater than 8 for all samples. Ribosomal RNA was depleted using the NEBNext® rRNA Depletion Kit v2 (New England Biolabs®) according to the manufacturer's instructions. Following depletion, the remaining RNA was quantified using a Qubit™ 4 Fluorometer (Thermo Fisher Scientific®). Library preparation was performed with 50 ng of ribo-depleted RNA using the KAPA RNA HyperPrep Kit (Roche®). The final libraries underwent quality control on the TapeStation system and were quantified using the Qubit™ system to ensure optimal concentration and fragment size.

The samples were sequenced on an Illumina® NextSeq 2000 platform. Sequencing was performed using a P1 flow cell with 2 × 150 bp paired-end reagent.

### 2.4 HLA Genotyping

HLA genotyping of the cell lines was performed using Next Generation Sequencing (NGSgo kit, GenDX, Utrecht, The Netherlands), on Illumina MiSeq platform (Illumina, San Diego, California). Sequences were analyzed using NGSEngine software (v. 2.21.0.20156, according to version 3.43 of the IPD-IMGT/HLA Database).

### 2.5 Immunopeptidomic analyses

#### 2.5.1 Immunopeptidome purification

CaptivA sepharose beads (Repligen, Waltham, Mass. USA) were covalently conjugated to 10 mg/mL W6/32 (pan-anti-HLA-I) monoclonal antibody (BioXcell) using DMP as previously described. [20] Snap frozen cells samples were resuspended in 1 mL lysis buffer (0.5% (vol/vol) NP-40, 50 mM Tris, pH 8.0, 150 mM NaCl), and protease inhibitor cocktail (Complete Protease Inhibitor from Roche 200 mM Iodoacetamide) and rotated on ice for 30 min. Homogenates were clarified for 10 min at 2000 g, 4 °C and then for a further 60 min at 13,500 g, 4 °C. 2 mg of anti-HLA-I-conjugated beads were added to the clarified supernatants and incubated with constant agitation for 2 h at 4 °C. The captured HLA-I/β2microglobulin/immunopeptide complex on the beads was washed sequentially with 10 column volumes of low (isotonic, 0.15 M NaCl) and high (hypertonic, 0.45 M NaCl) TBS washes prior to elution in 10% acetic acid and dried under vacuum. Column eluate was diluted with 0.5 volumes of 0.1% TFA and then applied to tC18 reverse-phase columns (Waters, 500 mg sorbent/column). The columns were rinsed with 10 column volumes of 0.1% TFA and then the peptides were eluted in acetonitrile 28%. Eluate was dried using a centrifugal evaporator and re-suspended in 0.1% formic acid.

#### 2.5.2 LC-MS Acquisition - Orbitrap Astral

Peptides (between 5 and 10 μL loading volume) were loaded onto 75 μm × 25 cm nanoLC column (Aurora C18 ultimate, particle size 1.6 μm, 120 Å; IonOpticks) and were separated using two gradients, 122 min for HLA—I. The following settings were used: 0 min at 2% B, 1 min 5.0% B at 500 nL/min, 0.1 min 5.0% B at 200 nL/min flow rate, 110 min 35% B, 2 min 70% B, 4 min 99% B, 0.1 min increase flowrate at 500 nL/min 99% B and maintain until 122 min. Hereafter, the settings for the 72-min method: 0 min at 2% B, 1 min 5% B at 500 nL/min, 0.1 min 5% B at 200 nL/min flow rate, 60 min 35% B, 2 min 70% B, 4 min 99% B, 0.1 min increase flow rate at 500 nL/min 99% B and maintain until 72 min with phase A 0.1% formic acid in water and phase B 80% acetonitrile 19.9% water 0.1% formic acid.

The peptides eluted from the column were analyzed on an Orbitrap Astral (Thermo Fisher Scientific). Interfaced with FAIMS for HLA—I, 3 compensation voltage (CV) experiments were done (−20, −40 and -60 V) with outer electrode temperature set at 90 °C. MS1 spectra were collected in the *m*/*z* range between 350 and 800 Th at a 120,000-resolution using the Orbitrap for −40 & -60 V CV and 700 to 1500 Th for −20 CV experiment with the following settings: (i) Automatic gain control target: 300% and (ii) maximum injection time: 100 ms. MS2 spectra were collected in the m/z range between 110 and 1500 Th using the Astral analyzer with the following settings: (i) Automatic gain control target: 30%, (ii) maximum injection time: 100 ms, (iii) normalized collision energy: 29%, (iv) isolation width: 2 Th for experiment we filter for MS2 on charge state 1 to 4.

#### 2.5.3 Data analysis for immunopeptidomics

Raw spectrum files were analyzed using Proteome Discoverer 3.2 for ARDIA precursor and associated product ion peak lists which were searched against a UniProt database (20,350 entries, 06/2025) appended and with the full sequences for mutated proteins. A contaminant database (120 entries) in non-specific digest mode was applied. Parent mass error tolerance was set at 10 ppm and fragment mass error tolerance at 0.02 Da. Variable modifications were set for methionine oxidation (15.99 Da) and carboxyamidomethylation (57.02 Da) of cysteine. A maximum of three variable modifications per peptide were set. The false discovery rate (FDR) was estimated with decoy-fusion database searches and were filtered to 1% FDR with Inferys rescoring enabled.

### 2.6 Flow cytometry

Cells were harvested and washed twice with phosphate-buffered saline (PBS). Cell concentration was adjusted to 1 × 10^6^ cells/mL in PBS and cells were incubated with viobility dye (VioGreen, Miltenyi Biotec) for 20 min in the dark. Cells were then washed twice with FACS buffer (0,1% BSA, 0,5 mM EDTA), incubated with FcR blocking reagent (Miltenyi Biotec) for 15 min at 4 °C and washed twice with FACS buffer. Cells were then incubated with fluorescent antibodies for 20 min at 4 °C and washed twice with FACS buffer. REA anti-HLA-ABC PE, REA anti-HLA-A*02:01 PerCP-Vio700, REA anti-IgG1 isotype control PE and REA anti-IgG1 isotype control PerCP-Vio700 antibodies were purchased from Miltenyi Biotec. Data acquisition was performed using a MACSQuant flow cytometer (Miltenyi Biotec). A minimum of 10,000 events were acquired for each sample. Data analysis was carried out using MACSQuantify software (Miltenyi Biotec). The gating strategy included an initial selection of the cell population of interest based on forward scatter (FSC) and side scatter (SSC) parameters, followed by doublet exclusion using FSC-A/FSC-H discrimination and dead cell exclusion based on viability staining. Marker expression was subsequently quantified as mean fluorescence intensity (MFI). Compensation settings were established using single-stained controls to correct for spectral overlap between fluorochromes. All experiments were performed in at least three independent biological replicates.

### 2.7 Bioinformatic analyses of transcriptomic data

#### 2.7.1 Data processing

Raw microarray data (CEL files) were downloaded from ArrayExpress, cleaned, quality-checked using arrayQualityMetrics (version 3.62.0) and normalized with the Robust Multi-Array Average (RMA) method (oligo version 1.70.0). RMA normalization consists of background noise correction to estimate real signal, followed by quantile normalization to obtain intensity distributions comparable between samples and finally summarizing probes in one expression value per gene expressed in log2 to stabilize variance. [21] Probe annotation was performed using hugene20sttranscriptcluster.db (version 8.8.0) and retaining one probe set per gene.

The raw RNA-seq FASTQ files generated from CHS cell lines were first processed using FastQC (version 0.11.9), and quality reports were aggregated with MultiQC (version 1.12). Adapter trimming and removal of low-quality bases were performed using Cutadapt (version 3.5). Cleaned reads were aligned to the human reference genome (GRCh38) with the STAR aligner (version 2.7.10a) using default parameters and the two-pass mapping strategy. Gene-level quantification was performed using FeatureCounts (version 2.0.3). For visualization and comparative expression analyses, counts were converted to transcripts per million (TPM) using a Python script (Python 3.12.11). To identify coding mutations present in the cell lines, aligned reads were also processed for single nucleotide polymorphism (SNP) identification, following GATK (version 4.6.2.0) standard steps (sorting, indexing, and duplicate marking), enabling detection of expressed sequence variants.

#### 2.7.2 Clustering analyses

To explore transcriptional differences across patients, unsupervised clustering analyses were performed. Hierarchical clustering using the complete linkage method and Euclidean distance was applied to expression values. Heatmaps and dendrograms were generated to visualize clustering using ComplexHeatmap (version 2.22.0) to identify gene and sample groups.

#### 2.7.3 Survival analyses

Univariate Cox proportional hazards models (R package survival, version 3.8–3) were used to evaluate associations between gene expression and survival. Hierarchical clustering was performed to identify patient subgroups based on the expression of genes significantly associated with survival, and Kaplan-Meier survival curves were generated. Survival differences between groups were evaluated using the log-rank test.

#### 2.7.4 CTA expression and immunophenotype

CTA expression levels in CHS patients were analyzed from the normalized microarray dataset. Expression values were transformed into z-scores to allow cross-sample comparison to identify groups. [22]

CTA expression in normal human tissues using GTEx-HPA consensus RNA-seq data allowed us to evaluate tissue expression. For each gene, the mean TPM value per tissue was computed and log10-transformed for visualization. CTA expression profiles in normal tissues and CHS samples were displayed using scatter plots to show tumor-specific CTA expression.

Immune cell infiltration in CHS samples was estimated using immune gene signatures from Bindea et al. [19] Correlations between CTA expression and immunophenotypes were assessed using Pearson correlation coefficients. For this analysis, the global mean expression of CTA genes was first computed, and these values were then correlated with the corresponding immune cell infiltration means. These analyses enabled investigation of the relationship between CTA expression and the level of immune cell infiltration.

### 2.8 Bioinformatic analyses of immunopeptidomic data

To compare the repertoires of HLA-presented peptides from JJ012 and CH2879 cell lines, peptide lists were compared against IEDB-annotated peptides and visualized using Venn diagrams to identify novel peptides.

NetMHC (version 4.0) was used to predict the binding affinity of novel identified peptides to the HLA alleles expressed by each cell line. Predictions were used to complement MS-based identifications and to determine which CTA-derived peptides could be stably presented by the HLA molecules of each cell line.

To predict the potential immunogenicity of each peptide, the IEDB immunogenicity prediction tool was used. These models estimate the probability of inducing a T cell response based on physicochemical and sequence features. Predictions were performed for CTA peptides from IEDB and our dataset.

Immunopeptidomic results were integrated with transcriptomic expression data to prioritize clinically relevant CTA candidates. For CTAs significantly associated with survival, TPM values from cell lines RNA-seq, were matched with protein abundance, immunophenotype, and predicted immunogenicity scores.

This integrative approach enabled the identification of CTA-derived peptides that were both transcribed, HLA-presented, and potentially immunogenic, representing promising antigenic targets in CHS.

## 3 Results

### 3.1 Cancer testis antigen gene expression is associated with patient outcome in CHS

**Fig. 1:**
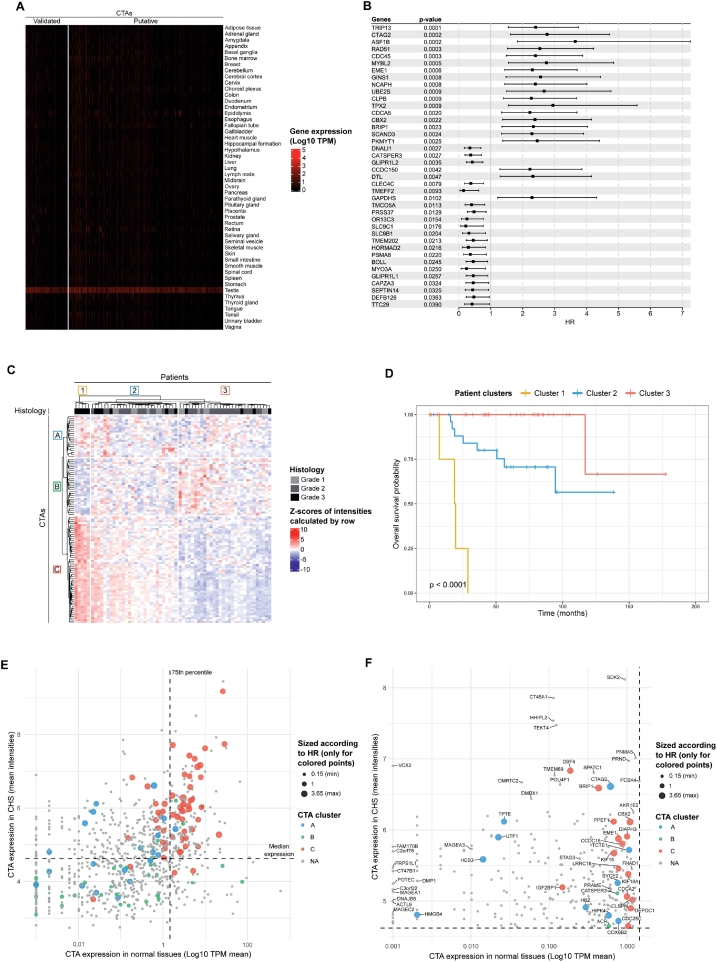
Association between CTA expression and the outcome of patients with conventional CHS. (**A**) Heatmap showing the expression levels of validated and putative CTAs across normal human tissues (GTEx and HPA consensus datasets). Expression values are displayed as log10(TPM); (**B**) Forest plot of the CTAs significantly associated with overall survival in conventional CHS (univariable Cox proportional hazards model, *p* < 0.05). Hazard ratios (HR) and 95% confidence intervals are shown for the 20 CTAs with the highest HR and the 20 with the lowest HR; (**C**) Heatmap of CTAs significantly associated with the overall survival of patients with conventional CHS (*n* = 63). Hierarchical clustering of patients and CTAs reveals three CTA expression clusters and three patient clusters. The tumor grade is annotated; (**D**) Kaplan-Meier curves corresponding to the patient clusters identified in panel C. Significant differences in survival were assessed using the log-rank test; (**E**) Scatter plot displaying the expression of CTAs in conventional CHS (y-axis, expressed as mean intensity) versus normal tissues (x-axis expressed as log10-transformed mean TPM). Dashed lines indicate the 75th percentile of CTA expression in normal tissues (vertical line) and the median CTA expression in CHS samples (horizontal line), used to stratify CTAs according to relative tumor vs normal expression levels. Selected CTAs of particular interest are annotated. Point size represents the HR for overall survival, and colors correspond to CTA clusters identified in panel C. (F) Scatter plot from E with a focus on points with intensity above the median and below the 75th percentile.

Tumor antigens restricted to cancer cells and associated with a poor prognosis represent highly attractive therapeutic targets. To identify clinically relevant candidate targets in CHS, correlation analyses were performed between the outcome of CHS patients and the expression genes showing a CTA profile based on public transcriptomic datasets. [18] Consistently with their expected germline-restricted pattern of expression, selected genes were not expressed, or at very low levels, in healthy tissues compared to the testis (Fig. 1A). Cox regression analysis identified the expression of 108 CTAs significantly correlated with overall survival of CHS patients (Fig. 1B). Based on *p*-values, TRIP13 and CTAG2 were the top two genes associated with short survival probability whereas DNALI1 and CATSPER3 were the top two CTAs correlating with the highest survival probability. Unsupervised hierarchical clustering analysis was performed to identify whether CHS patients could be stratified based on co-expressed gene signatures using the CTAs associated with overall survival only. This analysis revealed three groups of patients with distinct CTA expression profiles strongly correlated with the probability of overall survival which were mainly driven by CTAs of clusters B and C (Fig. 1C and D). High expression of CTAs of cluster C was associated with worse prognosis whereas high expression of CTAs of cluster B correlated with good overall survival probability. Although, the expression of CTAs of cluster A appeared more heterogeneous across the three patient groups, a high expression of these CTAs was noticed in the worst prognosis patients (cluster 1) which was comprised exclusively of grade 3 tumors. Gene ontology analyses showed that cluster C was remarkably composed of genes involved in cell proliferation processes which is consistent with their expression profile in high grade CHS and poor prognosis value (Fig. S1A–C). Interestingly, our CTA gene expression signatures were more accurate than the histological grade to predict the overall survival of conventional CHS patients (Fig. S1D). These findings demonstrate that CTA expression signatures can stratify CHS patients according to survival risk and may provide valuable prognostic insights.

To further highlight the best therapeutic candidate targets exhibiting a CHS specific expression profile versus healthy tissues, the CTA expression in normal samples versus CHS samples was displayed on a scatter plot (Fig. 1E–F). CTAs of cluster C showed the highest relative expression level in healthy tissues (above 75th percentile) compared to CTAs of cluster A. CTAs from clusters A and C were more expressed than CTAs of cluster B in CHS tumors. Well-characterized and already therapeutically exploited CTAs such as CTAG2, PRAME, MAGEA1 or MAGEA3 were found expressed in CHS and are shown as reference genes. SDK2, CT45A1, HHIPL2 and TEKT4 were the highest expressed CTAs in CHS. Among the poor prognosis genes, DBF4, CTAG2, BRIP1, TPTE, PPEF1, CBX2, DIAPH3, EME1, CCDC15, TCTE1 and HES3 were expressed at high levels in CHS and at relatively low levels in healthy tissues (below 75th percentile). VCX2, FAM170B, C2orf78, PRPS1L1, CT47B1, POTEC, C3orf22, MAGEA1, DNAJB8, ACTL9 and MAGEC2 were expressed in CHS but not detected in normal tissues. This analysis reveals that the MAGE family, PRAME and CTAG2, against which existing TCR-T cells therapies have been developed, may be relevant candidate targets in CHS. Beyond these well-known CTAs, these data also highlight original genes having a favorable expression and prognostic profile as therapeutic targets in CHS, based on transcriptomic data.

### 3.2 Integrated transcriptomic and immunopeptidome analyses identify CTA-derived pHLA in CHS

**Fig. 2:**
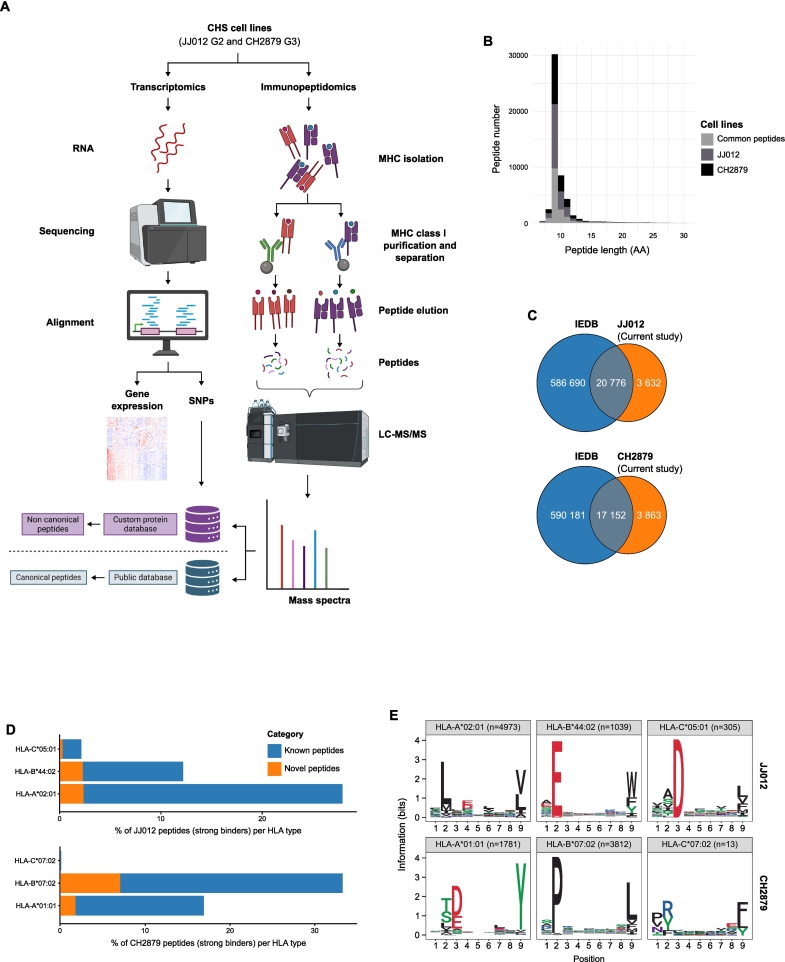
Integrated analysis identifies CTA-derived pHLA presented in CHS. (**A**) Schematic representation of the integrated workflow combining transcriptomic and immunopeptidomic analyses in grade 2 (JJ012) and grade 3 (CH2879) conventional CHS cell lines. RNA sequencing was performed to quantify gene expression and to characterize SNPs. HLA class-I were isolated, purified, and eluted and the repertoire of presented peptides was subsequently analyzed by LC-MS/MS. Canonical and non-canonical peptides were assigned using public and custom protein databases generated from the SNPs identification results; (**B**) Distribution of peptide lengths identified by LC-MS/MS in JJ012 and CH2879 cell lines; (**C**) Venn diagrams showing the overlap between peptides identified in this study and peptides found in the IEDB database for each cell line for peptides with length between 8 and 11 AA; (**D**) Percentage of IEDB known peptides versus novel identified peptides in CHS predicted as strong binders for each HLA allele expressed in JJ012 and CH2879 and determined by NGS; (**E**) Sequence logos for 9-mer peptides presented by the HLA alleles of each cell line, illustrating binding motifs.

To identify the CTAs expressed at protein level and to characterize the CTA-derived pHLA presented in CHS, an integrated transcriptomic and immunopeptidomic analysis was performed in two CHS cell lines (Fig. 2A). Peptide identification was enhanced using a house customized protein databases incorporating RNA-seq-derived variants, enabling the detection of both canonical and non-canonical CTA-derived pHLA. This integrative strategy provided a comprehensive overview of CTA expression and antigen presentation in CHS and allowed the identification of CTAs that are translated and effectively presented on HLA molecules. The CTA expression levels in cell lines and CHS samples isolated from patients were significantly correlated, demonstrating the clinical relevance of these models to identify CTA-related therapeutic targets (Fig. S2).

The immunopeptidomic profiling identified a broad repertoire of class-I pHLA, with a predominant length distribution centered on 9–11 amino acids in both models (Fig. 2B), consistent with the expected length of HLA class I antigens. Comparative analysis revealed both shared and cell line-specific immunopeptidomes, with novel peptides uniquely detected in each model. Comparison of the identified peptides with the IEDB database showed that 3632 peptides in JJ012 and 3863 peptides in CH2879 had not been previously reported, highlighting the presence of a substantial fraction of novel antigens in both cell lines (Fig. 2C). To identify allele-specific and novel CTA-derived peptides, HLA binding prediction analyses were performed based on HLA genotypes and peptide sequences using netMHC. Genotyping analyses determined the JJ012 model expressed HLA-A*02:01, B*44:02 and C*05:01 alleles whereas the CH2879 expresses HLA-A*01:01, B*07:02 and C*07:02 alleles. Both known- and novel-peptides included a significant proportion of strong predicted binders across the expressed HLA alleles (Fig. 2D). Peptide presentation in JJ012 was predominantly restricted by HLA-A*02:01, whereas HLA-A*01:01 was dominant in CH2879, in agreement with the respective HLA typing of each cell line. Sequence logo analyses of 9-mer peptides revealed allele-specific binding motifs that were consistent with established HLA anchor residue preferences, further validating the accuracy of peptide assignment and HLA restriction (Fig. 2E).

**Fig. 3:**
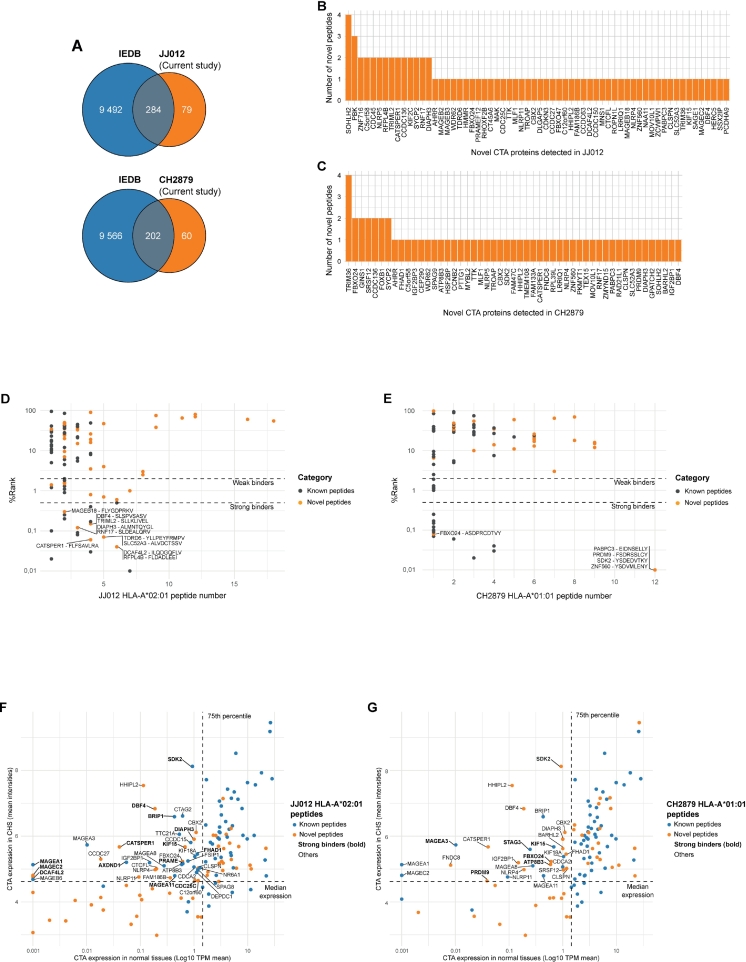
Identification of novel CTA-derived peptides in CHS models. (**A**) Venn diagrams showing the overlap between CTA-derived peptides detected in this study and those known in IEDB; (**B—C**) Bar plots representing the number of novel CTA-derived peptides identified in JJ012 and CH2879; (**D-E**) Scatter plots showing the peptide number and predicted binding affinity (%Rank from netMHC) for the dominant HLA alleles in each cell line (HLA-A*02:01 in JJ012 and HLA-A*01:01 in CH2879); (**F-G**) Scatter plot (adapted from Fig. 1E) illustrating CTA-derived pHLA expression presented by CHS cells. Gene names in bold correspond to CTAs giving rise to peptides predicted as strong binders to HLA-A*02:01 (F) or HLA-A*01:01 (G). Colors indicate whether the peptides were previously reported in IEDB or newly identified in this study.

363 CTA-derived peptides in JJ012 and 262 in CH2879 were identified, including 79 and 60 novel peptides respectively, with peptides derived from the MAGE family proteins (Fig. 3A, B and C). Some genes, such as SOHLH2, PBK, and TRIM36, generated multiple newly detected peptides, showing that these genes can generate multiple novel tumor-associated epitopes. The prediction analyses of HLA binding affinity demonstrated that these novel CTA-derived peptides spanned a wide range of %Rank values, including strong predicted binders for the dominant HLA alleles HLA-A*02:01 and HLA-A*01:01 expressed in each cell line (Fig. 3D–E).

Among the CTAs having a favorable expression profile as therapeutic targets, pHLA from the well-characterized CTAs such as CTAG2, PRAME, MAGEA1 and MAGEA3 were found in CHS cell lines and confirmed the translation of these CTAs in CHS models. The identified pHLA sequences correspond to antigenic sequences targeted by existing TCRs (Supplementary data 1) which rationalize the relevance of exploiting these TCRs in CHS using TCR-T cell therapy. Interestingly, other already described pHLA being strong predicted binders were immunoprecipitated from CTAs such as SDK2, BRIP1, AXDND1, FHAD1, MAGEA11, STAG3 and KIF15. In addition, we identified novel strong predicted binders for DBF4, DIAPH3, KIF15, CDC25C, MAGEC2, DCAF4L2 in the HLA-A*02:01 positive JJ012 cell line and SDK2, FBXO24, ATP8B3 and PRDM9 in the HLA-A*01:01 positive CH2879 cells (Fig. 3F–G).

**Fig. 4:**
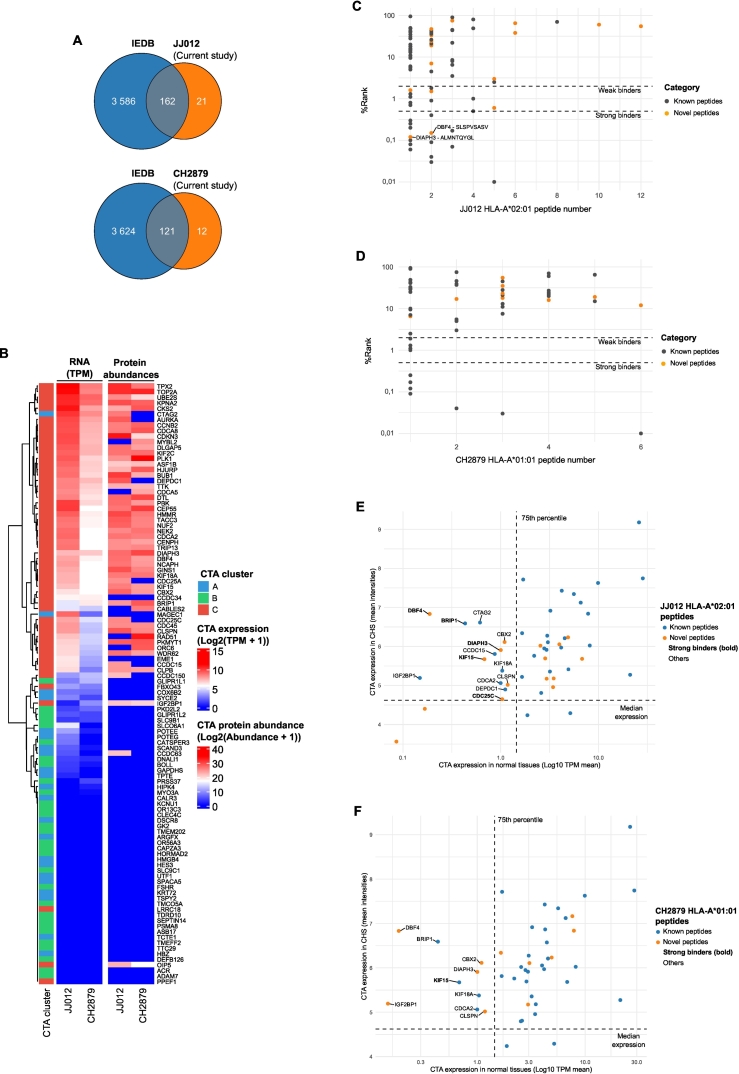
Focus on novel CTA-derived peptides significantly associated with overall survival in CHS models. (**A**) Venn diagrams showing the overlap between CTA-derived peptides significantly associated with overall survival detected in this study and those known in IEDB; (**B**) Heatmap showing the expression of significantly associated with overall survival at the RNA level (TPM) and their corresponding peptide abundances detected by immunopeptidomic analyses in JJ012 and CH2879 cell lines. The annotation of CTA prognosis clusters identified in CHS patients is indicated. Colors represent log2 values (TPM + 1 for RNA, abundance +1 for peptides); (**C—D**) Scatter plots showing the peptide number and predicted binding affinity (%Rank from netMHC) for the dominant HLA alleles in each cell line (HLA-A*02:01 in JJ012 and HLA-A*01:01 in CH2879); (***E*-F**) Scatter plots (adapted from Fig. 1E) illustrating CTA-derived peptides significantly associated with overall survival expression presented by CHS cells. Gene names in bold correspond to CTAs giving rise to peptides predicted as strong binders to HLA-A*02:01 (F) or HLA-A*01:01 (G). Colors indicate whether the peptides were previously reported in IEDB or newly identified in this study.

Among the CTAs associated with the overall survival of patients with CHS, 183 pHLA in JJ012 and 133 pHLA in CH2879 were identified, including 21 and 12 novel peptides respectively (Fig. 4A). As shown by the quantification of the protein abundance, most of the detected peptides were derived from CTAs of cluster C, only CTAG2 and CCDC63 from cluster A were presented and there was no peptide immunoprecipitated from CTAs of cluster B (Fig. 4B). These results indicate that most of the poor prognosis CTAs identified in CHS patients are presented in JJ012 and CH2879, supporting the relevance of these models. Interestingly, the global expression profile of the prognosis CTA was similar at the transcriptomic level in the JJ012 and CH2879 cell lines and was associated with the protein abundance. The prediction analyses of HLA binding affinity highlighted strong predicted binders derived from these CTAs for the dominant HLA alleles (Fig. 4C and D). Among these CTAs, peptides from DBF4, BRIP1, DIAPH3, KIF15, and CDC25C, which were identified as clinically relevant, were presented in the CHS cell lines (Fig. 4E and F). Detailed lists of novel peptides identified in JJ012 and CH2879 are provided in (Supplementary data 2).

### 3.3 Expression of prognosis CTA signatures is associated with the tumor immune infiltration in CHS

**Fig. 5:**
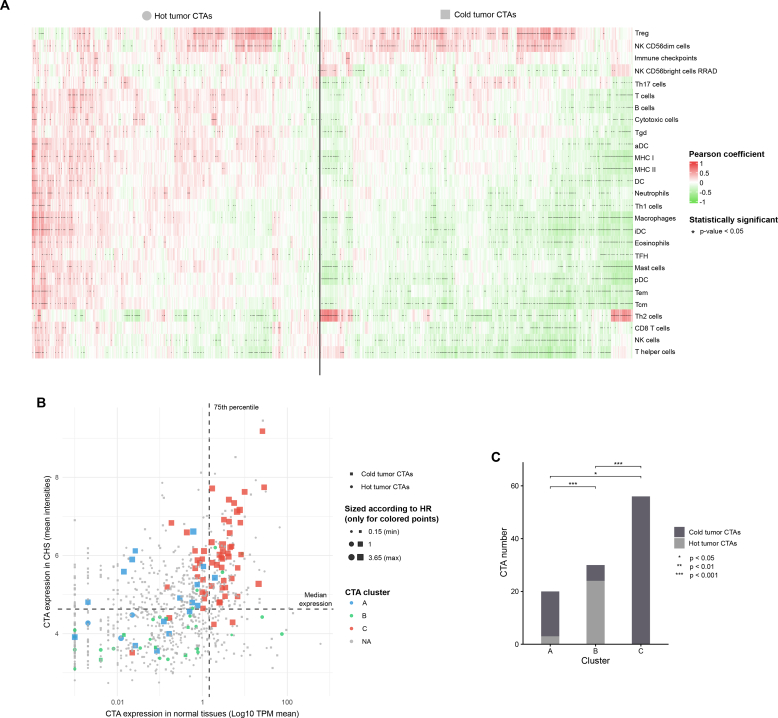
Relationship between CTA expression and tumor immune phenotype. (**A**) Pearson correlation heatmap between CTA expression and immune gene expression signatures in conventional CHS. Significant correlations (p < 0.05) are marked with an asterisk. Hierarchical clustering separates CTAs into two major immune phenotypes (hot tumor CTAs and cold tumor CTAs); (**B**) Scatter plot from Fig. 1E annotated with hot and cold tumor CTAs using different point shapes; (**C**) Bar plot showing the distribution of hot tumor CTAs and cold tumor CTAs across clusters. Statistical significance was assessed using pairwise Fisher's exact tests with Benjamini-Hochberg (BH) correction.

Since the tumor immune phenotype has been associated with the clinical outcome in several cancers including CHS [23] and that the presentation of CTA-derived peptides may modulate the antitumor immune functions, we investigated the relationship between CTA expression and the immune landscape in conventional CHS. Correlations analyses were performed between CTA expression levels and a panel of immune-related signatures across CHS patient using a gene expression a deconvolution method [19], [24]. Pearson analysis revealed heterogeneous associations with both positive and negative correlations observed between the expression of CTAs and the immune signatures (Fig. 5A). Hierarchical clustering analyses based on these correlations separated CTAs into two major immune-associated phenotypes, referred to as immunogically hot- versus cold-associated CTAs. CTAs associated with the hot phenotype were positively correlated with immune infiltration and activation signatures, including T cells, cytotoxic cells, and antigen presentation machinery, whereas cold-associated CTAs displayed weak or negative associations with these immune features. The integration of the immune-associated classifications into the previously generated expression-outcome scatter plot revealed that prognosis CTAs of clusters A and C were mostly cold tumor CTAs whereas CTAs of cluster B were mostly classified as hot tumor CTAs (Fig. 5B and C). Together, these observations indicate clear associations between the CTA expression signature, the level of immune infiltration and the clinical outcome. The classification of the CTAs of clusters A and C, expressed at relatively high level, as cold tumor CTAs suggest that other factors than the level of CTA expression may be involved in determining the tumor immune phenotype in CHS.

### 3.4 CTA immunogenicity is not associated with the level of tumor immune infiltration in CHS

**Fig. 6:**
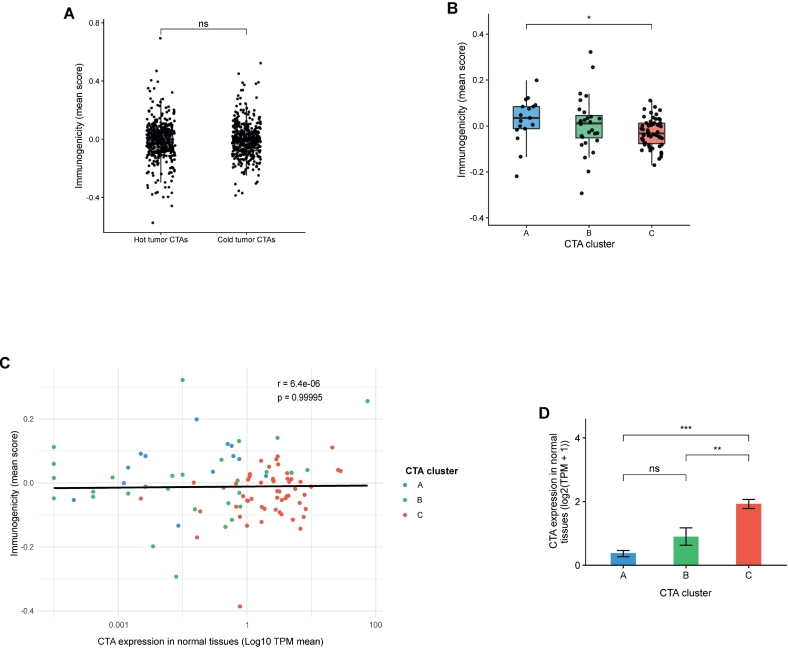
CTA expression and immunogenicity prediction. (**A**) Box plot showing the mean predicted immunogenicity score for each CTA (IEDB prediction tool), stratified by hot or cold tumor CTAs. Statistical significance was assessed using the Wilcoxon rank-sum test; (**B**) Comparison of mean predicted immunogenicity scores between hot and cold clusters (Wilcoxon rank-sum test); (**C**) Scatter plot showing the distribution of hot and cold tumor CTAs according to their expression profile and prognostic cluster; (**D**) Box plot showing the mean expression per cluster (Wilcoxon rank-sum test with BH correction).

Beyond the expression of CTAs in tumor cells, the immunogenicity of pHLA is another important parameter which could impact the level of immune cell infiltration in tumors. The association between the immune classification of CTAs and their predicted immunogenicity was evaluated. CTAs classified within the hot and cold clusters did not show any significant difference in mean predicted immunogenicity scores (Fig. 6A). Considering the prognosis CTAs only, the predicted immunogenicity of CTAs in cluster B, which were mostly classified as hot tumor CTAs, was surprisingly not higher than the other CTA clusters (Fig. 6B). However, the averaged predicted immunogenicity was lower in CTAs from cluster C than those in cluster A. CTAs in cluster C being expressed at higher levels in normal tissues than CTAs of cluster A (Fig. 6C), we evaluated whether CTA immunogenicity was associated with the expression level of CTAs in normal tissues. Linear regression analysis showed no significant correlation between mean predicted immunogenicity scores and mean CTA expression levels in normal tissues (Fig. 6C and D). This data suggests that CTA immunogenicity in CHS is not driven by physiological expression in normal tissues, reinforcing their relevance as tumor-associated antigens.

### 3.5 INFγ stimulation reveals additional targetable pHLA in CHS cells

**Fig. 7:**
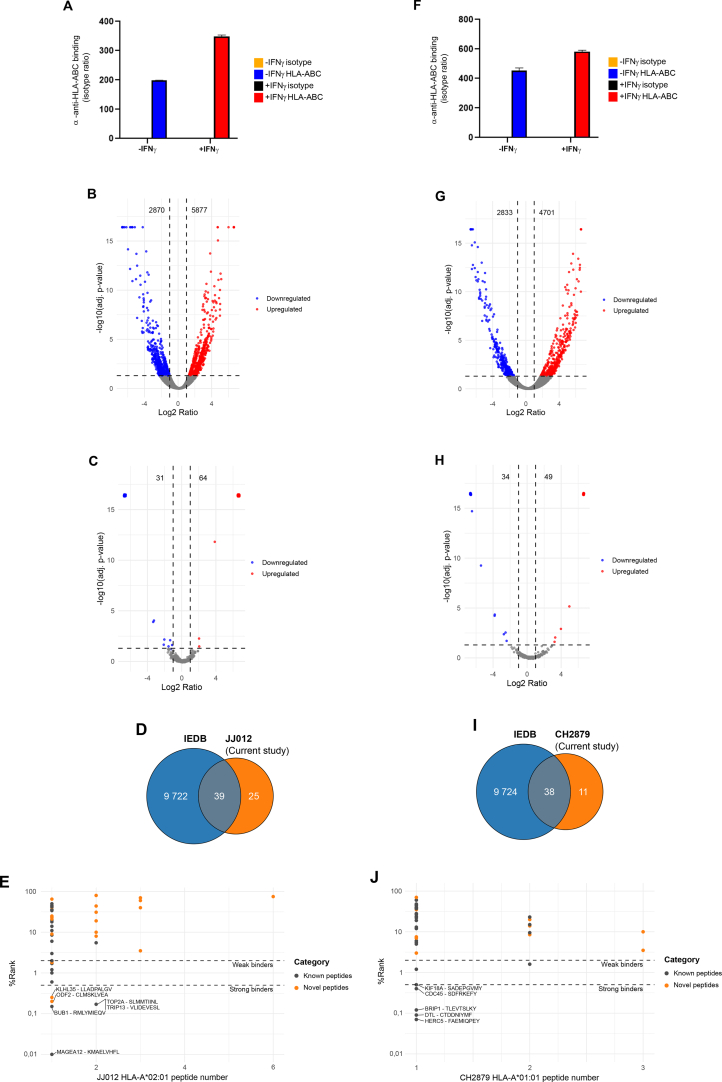
IFNγ-induced ligandome in CHS cell lines. (**A, F**) Bar plot shows the quantification of HLA class I surface levels under isotype or IFNγ conditions in flow cytometry for JJ012 (A) and CH2879 (F); (**B, C; G, H**) Volcano plots display differential peptide presentations following IFNγ treatment: all detected peptides (B and G) and CTA-derived peptides only (C and H). Peptides significantly upregulated are shown in red and downregulated in blue; (**D, I**) Venn diagrams showing the overlap between CTA-derived peptides significantly upregulated detected in this study and those known in IEDB for JJ012 (D) and CH2879 (I); (**E, J**) Scatter plots showing the peptide number and predicted binding affinity (%rank from netMHC) for the dominant HLA alleles in each cell line (HLA-A*02:01 in JJ012 (E) and HLA-A*01:01 in CH2879 (J)). (For interpretation of the references to color in this figure legend, the reader is referred to the web version of this article.)

IFNγ can modify the immunopeptidome landscape through the stimulation of antigen presentation machinery and the expression of HLA class I in cancer cells. To assess whether IFNγ could lead to the presentation of additional targetable pHLA in CHS, the immunopeptidome was compared in JJ012 and CH2879 cell lines stimulated or not with IFNγ. Flow cytometry analyses showed that HLA class-I was expressed and increased upon IFNγ stimulation as expected in both models (Fig. 7A and F). In lines with the genotyping data, we detected the expression of HLA: A*02:01 in JJ012 but not in CH2879, (Fig. S3).

Comparative immunopeptidomic analyses under basal and IFNγ-treated conditions revealed remodeling of the ligandome upon IFNγ exposure, with 5880 and 4709 peptides significantly upregulated in JJ012 and CH2879 respectively (Fig. 7B and G). Focusing specifically on CTA-derived peptides, IFNγ stimulated the presentation of 64 (25 novel) and 49 (11 novel) pHLA in JJ012 and CH2879 respectively (Fig. 7C, D, H and I). Strong predicted pHLA binders were identified for the HLA-A*02:01 allele in JJ012 including two novel peptides from CTA genes KLHL35 and ODF2. IFNγ stimulated the presentation of the peptide KMAELVHFL from CTA gene MAGE12 which is targetable with the TCR A31D5-B32B5. In CH2879, strong predicted pHLA binders from CTAs were identified including BRIP1 for the HLA-A*01:01 allele. These changes indicate that IFNγ not only enhances antigen presentation capacity but also modify the repertoire of pHLA among which some exhibit a relevant expression profile in CHS patients.

## 4 Discussion

CHS remains one of the most therapeutically challenging bone malignancies due to its resistance to conventional treatments highlighting the urgent need for novel therapeutic option including immunotherapy-based strategies. This study provides the first integrated characterization of CTAs and their associated pHLA in CHS, combining transcriptomic profiling with immunopeptidomic analyses of CHS tumors and models, offering novel and clinically relevant antigens for the development of targeted immunotherapy.

Transcriptomic analysis revealed a repertoire of CTAs expressed in CHS with limited expression in normal tissues. Hierarchical clustering identified distinct patient subgroups reflecting inter-patient heterogeneity, with a subset showing particularly high CTA expression. This heterogeneity has direct implications for patient stratification, as CTA-based therapeutic approaches may be most applicable to this high-expressing subgroup. Among the CTAs identified in CHS, PRAME, CTAG2 and MAGE-A family members (MAGEA1, MAGEA3, MAGEA12) stand out as compelling therapeutic targets due to their tumor associated expression across multiple solid tumors and confirmed pHLA presentation in CHS cell lines. [25], [26] PRAME, a well-characterized CTA regulating cell differentiation, growth, and apoptosis, has already been explored in phase I and II clinical trials involving peptide vaccines and TCR-engineered T cells in melanoma, ovarian cancer and synovial sarcoma, supporting its translational potential in CHS. [27], [28] A recent study confirmed the expression of PRAME in CHS sample by immunohistochemistry. [29] CTAG2 expression was associated with poor prognosis in CHS patients, supporting the relevance of testing previously proposed antigen-specific T cell therapies in CHS. [30] However, we did not detect the most widely exploited antigen (SLLMWITQC) HLA-A*02:01 peptide derived from CTAG2_157–165_ (identical to NY-ESO-1_157-165_ epitope) in the JJ012 cell line. MAGE-A family members share restricted expression in normal adult tissues and broad aberrant expression across several tumors, where overexpression has been linked to poor prognosis and oncogenic activity. [26], [31]

Despite their extensive exploration as therapeutic targets through peptide vaccines and TCR-transduced T cell strategies, MAGE-A-directed therapies have failed to demonstrate robust clinical efficacy mainly because of severe adverse effect linked to off target effects. [26], [32], [33] For example, the MAGE-A3 TCR (clone 9 W11) directed against MAGE-A3_112–120_ (KVAELVHFL) presented by the HLA-A*02:01 allele has shown to cross react against MAGE-A12_112–120_ (KMAELVHFL) and EPS8L2_339–347_ (SAAELVHFL), both presented by in the HLA-A*02:01 context. The expression of MAGE-A12 and EPS8L2 in the brain have been shown to explain the neurotoxic effects observed in patients treated with TCR-T-cells therapy using the 9 W11 clone and has limited the clinical use of this TCR. [26] MAGE-A3_282–290_ (SYVKVLHHM) presented in the HLA-A*02:01 positive JJ012 cell line upon IFNγ exposure. This peptide is known to bind the HLA-A*24:02 allele but was found to bind potentially to the HLA-A*02:01 allele with strong affinity in our predictive analysis. This result opens the perspective to target this epitope rather than MAGE-A3_112–120_ in HLA-A*02:01 positive CHS patients. Beyond MAGE-A3, our study identified HLA-A*02:01 epitopes from MAGE-A1_278–286_ (KVLEYVIKV) and MAGE-A1_161–169_ (CILESLFRA) which represent potential promising peptides to target in the MAGE-A family for further investigations in CHS.

Beyond established CTAs, our immunopeptidomic analysis identified peptides derived from BRIP1, DBF4, HHIPL2 and SDK2 as novel pHLA targets. BRIP1 (BRCA1 Interacting DNA Helicase 1), a DNA repair helicase involved in the Fanconi anemia pathway, is upregulated and associated with poor overall survival across multiple cancer types. [34] DBF4 (DBF4B-CDC7 Kinase Regulatory Subunit), the regulatory subunit of the DDK complex, is frequently overexpressed in melanoma, lung, gastric and hepatocellular cancers, where it drives proliferation, invasiveness and chemoresistance, and was confirmed as a negative prognostic factor in CHS in our analysis (HR = 1.9). [35] HHIPL2 (Hedgehog Interacting Protein Like 2), a positive regulator of the Sonic Hedgehog pathway, is overexpressed in lung cancer and associated with advanced stage and shorter survival in non-small cell lung carcinoma (NSCLC). [36] Its relevance in CHS is underscored by evidence that Hedgehog signaling actively contributes to tumor growth in this context, and that inhibition of this pathway reduces CHS cell proliferation in preclinical models. [37] SDK2 (Sidekick cell adhesion molecule 2), a cell adhesion molecule of the immunoglobulin superfamily, has been implicated in tumor biology through its potential role in regulating cell-cell interactions and metastatic behavior, although its precise function in cancer remains poorly defined. Since SDK2 is a transmembrane protein, its direct targeting could be prioritized, regardless of the SDK2-derived pHLA identified in our study. Together, the cell surface presentation of BRIP1-, DBF4-, HHIPL2-derived peptides and SDK2, combined with the tumor-associated expression profile of the gene in CHS and their frequent prognostic significance observed in other cancer types, supports their further investigation as targets in CHS and beyond.

Other CTAs including CT45A1, VCX2 and TEKT4 emerged as potential innovative targets based on their low expression in normal tissues and elevated tumor expression in CHS. CT45A1 has been implicated in proliferation and metastasis in bone sarcomas [38], VCX2 identified as a highly tumor-specific epigenetically inducible CTA in melanoma [39], and TEKT4 is implicated in tumorigenesis [40]. However, no HLA-A*02:01 or HLA-A*01:01 predicted peptides were detected for these antigens in our immunopeptidomic analysis, which limits their therapeutic potential through pHLA targeting.

CHS is characterized as an immunologically cold tumor with poor lymphocyte infiltration, though T cells are detectable at the tumor periphery and their presence correlates with improved survival. [23], [41], [42] Tumor size negatively correlates with CD8+ T cell infiltration, suggesting that immune effectors are excluded from the tumor core, potentially due to the high proteoglycan content of the cartilaginous matrix. [41], [43] In this context, approaches independent of endogenous immune infiltration such as antibody-drug conjugates, may represent promising strategies for CHS treatment. The efficacy of antibody-drug conjugate directed against pHLA was shown in preclinical models with the NY-ESO1_157–165_ /CTAG2_157–165_ TCR mimicking antibody conjugated to the anti-proliferative agent monomethyl auristatine E. [44]

Several limitations in this study warrant consideration. First, CHS is a rare disease and limited public transcriptomic and clinical datasets constrain statistical power. The absence of CHS in major repositories limits the validation of our finding in independent cohorts. Second, our immunopeptidomic analyses were conducted in cell line models, which may not fully recapitulate the antigen presentation landscape of primary tumors, particularly given the influence of the tumor microenvironment and tumor heterogeneity. Third, it should be noted that the HLA alleles covered in this study do not encompass full population diversity, which limits the generalizability of the identified pHLA targets. Fourth, the binding affinity predictions should be validated experimentally for the novel pHLA identified here. Future studies incorporating primary CHS tumor samples, patient-derived HLA typing, and ex vivo T cell stimulation assays will be required to validate the clinical relevance of the identified targets. Fifth, immune cell deconvolution from gene expression data allowed stratification of tumors by immune infiltration level, however this approach remains a computational surrogate pending histological validation.

Taken together, our findings provide the first comprehensive characterization of the CTA landscape and HLA class-I immunopeptidome in CHS, opening new perspectives for antigen-specific immunotherapies including adoptive T cell therapies or ADCs, laying the groundwork for future translational studies in this disease.

## CRediT authorship contribution statement

**Léa Rogue:** Writing – original draft, Visualization, Methodology, Formal analysis, Conceptualization. **Jean-Marc Monneuse:** Writing – review & editing, Writing – original draft, Methodology, Formal analysis. **Céline Béchon:** Methodology, Formal analysis. **Lola Cepero:** Writing – review & editing, Formal analysis. **Caroline Peyrode:** Writing – review & editing. **Sandrine Viala:** Methodology, Formal analysis. **Maud Privat:** Methodology. **Yannick Bidet:** Methodology. **Adrien Saliou:** Writing – review & editing, Supervision, Methodology. **Paul-Olivier Rouzaire:** Supervision, Methodology. **Elisabeth Miot-Noirault:** Writing – review & editing, Resources, Funding acquisition. **Florent Cachin:** Writing – review & editing, Resources, Project administration, Funding acquisition. **Aurélien Pommier:** Writing – review & editing, Writing – original draft, Visualization, Validation, Supervision, Project administration, Methodology, Funding acquisition, Formal analysis, Conceptualization.

## Funding

This research was funded by the CIR3 I-Site Excellence program from the Université Clermont Auvergne, grant RACImBOOST.

## Declaration of competing interest

The authors declare that they have no known competing financial interests or personal relationships that could have appeared to influence the work reported in this paper.

## Data Availability

The datasets analyzed in this study are publicly available under accession number E-MTAB-7264, from HPA (https://www.proteinatlas.org/humanproteome/tissue/data#consensus_tissues_rna) and from IEDB (https://www.iedb.org/database_export_v3.php). RNA-seq raw data from cell lines are available from the corresponding author upon reasonable request. The code required to reproduce the figures of this study is available at https://github.com/learogue/CHS_CTAs_bioinformatics and archived on Zenodo at https://doi.org/10.5281/zenodo.20322622.

## Appendix A Supplementary data

Supplementary material: pHLA differantially presented in response to INFγ in JJ012 and CH2879(Figure 7)
